# Transcription Factor Bcl11b Controls Effector and Memory CD8 T cell Fate Decision and Function during Poxvirus Infection

**DOI:** 10.3389/fimmu.2016.00425

**Published:** 2016-10-13

**Authors:** Georges Abboud, Jessica Stanfield, Vikas Tahiliani, Pritesh Desai, Tarun E. Hutchinson, Kyle J. Lorentsen, Jonathan J. Cho, Dorina Avram, Shahram Salek-Ardakani

**Affiliations:** ^1^Department of Pathology, Immunology and Laboratory Medicine, College of Medicine, University of Florida, Gainesville, FA, USA; ^2^Department of Medicine, Division of Pulmonary Medicine, College of Medicine, University of Florida, Gainesville, FA, USA

**Keywords:** T cells, transcription factors, BCL11b, memory, lung, viral, poxvirus

## Abstract

CD8^+^ T cells play an important role in host resistance to many viral infections, but the underlying transcriptional mechanisms governing their differentiation and functionality remain poorly defined. By using a highly virulent systemic and respiratory poxvirus infection in mice, we show that the transcription factor Bcl11b provides a dual trigger that sustains the clonal expansion of virus-specific effector CD8^+^ T cells, while simultaneously suppressing the expression of surface markers associated with short-lived effector cell (SLEC) differentiation. Additionally, we demonstrate that Bcl11b supports the acquisition of memory precursor effector cell (MPEC) phenotype and, thus, its absence causes near complete loss of lymphoid and lung-resident memory cells. Interestingly, despite having normal levels of T-bet and Eomesodermin, Bcl11b-deficient CD8^+^ T cells failed to execute effector differentiation needed for anti-viral cytokine production and degranulation, suggesting a non-redundant role of Bcl11b in regulation of this program. Thus, Bcl11b is a critical player in fate decision of SLECs and MPECs, as well as effector function and memory formation.

## Introduction

The murine model of vaccinia virus (VacV) infection has proven to be a powerful system for investigating immune responses to highly virulent viruses. Vaccinia is a large DNA virus that replicates in the cytoplasm of infected cells. Systemic (i.p., or i.v.) or respiratory inoculation of immunocompetent mice with the mouse adapted Western Reserve strain (VacV-WR) results in an acute infection during which the virus replicates very rapidly to high titers, causes strong and sustained inflammation, and disseminates to multiple lymphoid and non-lymphoid tissues within the host (1–4). Activated macrophages (5), natural killer (NK) cells (6, 7), and γδ T cells (8) play an important role in the initial control of VacV (9). Adaptive immune responses mediated by CD4^+^, CD8^+^, and B-lymphocytes take several days to develop and are necessary for complete clearance of virus from infected tissues (2, 10, 11). Upon reinfection, virus-specific memory CD8^+^ T cells traffic to the lung parenchyma and airway lumen, mediating rapid clearance and providing protective immunity against otherwise lethal challenges (1, 12, 13).

In recent years, we have defined some of the key co-stimulatory pathways required to program naïve VacV-specific CD8^+^ T cells with different antigen specificities to expand, differentiate, and survive to become memory cells (14, 15). Surprisingly, however, little is known about the underlying transcriptional mechanisms that govern the development of functional virus-specific effector and memory CD8^+^ T cells after VacV infection.

During lymphocytic choriomeningitis virus (LCMV) infection, two members of the T-box transcription factor family, T-bet and eomesodermin (Eomes), cooperate to induce expression of IFN-γ, perforin, and granzyme B by early effector CD8^+^ T cells (16). Interestingly, they also seem to have discrete functions (16). For example, high levels of T-bet have been associated with differentiation of short-lived effector cells (SLECs), which are defined by the upregulation of KLRG1 and the loss of IL-7Rα (CD127). Conversely, early expression of Eomes has been shown to favor the formation of memory precursor effector cells (MPECs) that express low levels of KLRG1 and increased levels of CD127 and IL-2 (17). Induction of T-bet in Ag-specific CD8^+^ T cells is mediated initially by TCR signaling and amplified by IL-12 (18, 19). The expression of Eomes appears to be induced subsequently to that of T-bet and can be amplified by IL-2, but suppressed by IL-12 (18, 20). Accordingly, IL-12 has been proposed to be a molecular switch between SLEC and MPEC cell fate decision by promoting high levels of T-bet expression while suppressing Eomes. Further studies have implicated numerous other transcription factors in SLEC vs. MPEC cell fate decision with Blimp-1, and Id2 favoring SLEC formation and Bcl-6, Tcf1, FOXO1, STAT3, and Id3 expression supporting the formation of MPECs (16). Thus, coordinated and dynamic expression of multiple transcription factors ensures formation of distinct effector and memory populations under various physiological conditions. Whether other transcription factors participate in overlapping or complimentary pathways in the differentiation of effector and memory CD8 T cell responses to viral pathogens *in vivo* is not well defined.

Bcl11b is a C2H2 zinc finger transcription factor known to function as both a transcriptional activator and repressor depending on its interacting partners (21). In T cells, Bcl11b expression begins in the DN2 state of thymocyte development and continues as thymocytes mature (22). Bcl11b is also expressed in mature CD4^+^ and CD8^+^ T cells (23–25) and innate lymphoid cells (26) as well as in regulatory T (Treg) cells (27) and invariant Natural Killer T (iNKT) cells in the thymus and periphery (28, 29). Our recent report suggested that priming of CD8^+^ T cells in lymphoid tissues is compromised in the absence of Bcl11b (24). After systemic infection with *Listeria monocytogenes*, Bcl11b was shown to be required for antigen (Ag)-dependent clonal expansion in the spleen (24). Furthermore, after respiratory influenza virus infection, the frequency of nucleoprotein (NP)- and polymerase complex PA subunit (PA)-specific effector Bcl11b-deficient CD8^+^ T cells in the mediastinal lymph nodes was significantly reduced (24). While these studies demonstrated that Bcl11b drives CD8^+^ T cell proliferation and effector function in the lymphoid compartment, little is known regarding the role of Bcl11b as potentially driving effector and/or memory CD8^+^ T cell differentiation in mucosal tissues. In the present study, we used a conditional knock out (Cko) mouse model to compare the requirement for Bcl11b in CD8^+^ T cell responses induced in the spleen and lung microenvironments after systemic and respiratory VacV infection. We report that the absence of Bcl11b during priming of CD8^+^ T cells has major phenotypic, cell fate, and functional consequence on the effector and memory cells generated during an immune response to VacV infection.

## Materials and Methods

### Mice

Eight- to 12-week-old female *Bcl11b*^*flox/flox*^/dLck-iCre^+^ mice on C57BL/6 background and wild-type littermate mice were bred and maintained at the University of Florida animal facility. *Bcl11b^flox/flox^*/dLck-iCre^+^ mice were previously described (23–25). This study was carried out in strict accordance with the recommendations in the Guide for the Care and Use of Laboratory Animals of the animal Welfare Act and the National Institutes of Health guidelines for the care and use of animals in the biomedical research. All animal protocols were approved by the Institutional Animal Care and Use Committee (IACUC) of the University of Florida, Gainesville (OLAW Assurance # A3377-01).

### Peptides and Tetramers

Vaccinia virus peptide epitopes used in this study were predicted and synthesized as described previously (30, 31). The VacV-WR peptide B8R_20–27_ (TSYKFESV) and A8R_189–196_ (ITYRFYLI) (30, 31) were purchased From A & A Labs (San Diego). MHC/peptide tetramers for the VacV Western Reserve (WR) epitope B8R (20-27; TSYKFESV)/H-2K^b^, which were conjugated to allophycocyanin, were obtained from the National Institutes of Health Tetramer Core facility (Emory University, Atlanta, GA, USA).

### Virus and Peptides

Vaccinia virus Western Reserve (VacV-WR) was purchased from the American Type Culture Collection (ATCC), grown in HeLa cells, and subsequently titered on VeroE6 cells as previously described (12).

### Virus Infections

Naïve mice were infected with 2.0 × 10^5^ plaque forming unit (PFU) VacV-WR by bilateral i.p. injections. For respiratory VacV-WR infection model, naïve mice were anesthetized by isoflurane inhalation and infected intranasally (i.n.) with 1.25 × 10^4^ PFU of VacV-WR, with daily measurements of body weight [as described before (1, 2)]. Body weight was calculated as percentage of the mean weight for each mouse on the day of challenge. Effector responses were analyzed between days 8 and 15 postinfection, while memory responses were analyzed 40 or more days after infection.

### VacV-Titer Assay

Tissues from individual mice were homogenized, and sonicated for 0.5 min with a pause every 10 s using an ultrasonic cleaner 1210 Branson (Danbury, CT, USA). Serial dilutions were made and the virus titers were then determined by plaque assay on confluent VeroE6 cells.

### Flow Cytometric and FACS Analysis

Preparation of cells, extracellular and intracellular staining, FACS data acquisition, and analysis were performed as described previously (1, 11, 32). Briefly, all tissues were aseptically removed from euthanized mice, and single-cell suspensions were prepared by mechanically dispersing the tissues through 70-μm cell strainers (Falcon BD Labware) into HBSS. Lung tissue was treated for 1 h at 37°C with 25°μg Librase TL (Roche) followed by treatment for 10 min at 4^o^C with 100°μM EDTA supplemented media. Following lysing RBC, splenocytes from infected mice were resuspended in RPMI 1640 medium (Life Technologies) supplemented with 10% FCS (Omega Scientific), 1% l-glutamine (Invitrogen), 100 mg/ml streptomycin, 100 U/ml penicillin, and 50 mM 2-ME (Sigma-Aldrich).

#### T Cell Subsets

Cells were washed with FACS buffer [phosphate buffered saline (PBS) and 2% FCS] and stained with anti-Fc II/III receptor monoclonal antibody 2.4G2 for 15 min at 4°C. After an additional FACS buffer wash, the following antibodies were incubated with the cells for 30 min at 4°C: CD4 (RM4-5, eBioscience), CD8α (53-6.7, eBioscience), CD3 (145-2C11, eBioscience), CD44 (IM7, eBiosience), and B8R tetramer (NIH tetramer facility) were used to determine total T cell and VacV-specific CD8 T cell subsets. Isolated spleen and lung B8R^+^ CD8 T cells from WT and *Bcl11b*^−/−^ mice were also stained for their surface expression of KLRG-1 (eBioscience) and IL-7Ra (eBioscience). All samples were acquired on a FACS LSR II or Canto II (BD Bioscience) and analyzed using FlowJo (Tree Star).

#### VacV-Specific Cytokine Production

One to 2 × 10^6^ cells were plated in round-bottom 96-well microtiter plates in 200 μl with medium or the indicated VacV peptides at 1 μg/ml for 1 h at 37°C. GolgiPlug (BD Biosciences) and anti-CD107α (1D4B, eBioscience) was then added to the cultures according to the manufacturer’s instructions and the incubation was continued for 8 h. Cells were stained with anti-CD8 (PerCP; 53-6.7) and CD62L (PE; MEL-14), followed by fixation with Cytofix/Cytoperm (BD Biosciences) for 20 min at 4°C. Fixed cells were subjected to intracellular cytokine staining in BD Perm/Wash buffer for 30 min at 4°C. Anti-IFN-γ (allophycocyanin; XMG1.2), anti-TNF (MP6-XT22; PE Cy7), and anti-IL-2 (JES6-5H4) were obtained from eBiosience and used at a 1/150 dilution. Samples were analyzed for their proportion of cytoplasmic cytokines after gating on CD8^+^CD62L^low^ T cells by a FACSCalibur flow cytometer using CellQuest (BD Biosciences) and FlowJo software (Tree Star).

### Statistical Analysis

Tests were performed using the Prism 5.0 software (GraphPad, San Diego, CA, USA). Statistics were done using two-tailed, unpaired Student’s *t*-test with 95% confidence intervals unless otherwise indicated. Unless otherwise indicated, data represent the mean ± SEM; *p* < 0.05 considered statistically significant.

## Results

### Requirement for Bcl11b in Primary VacV-Specific CD8^+^ T Cell Expansion in Lymphoid vs. Mucosal Compartment

Whether Bcl11b contributes to the development and maintenance of memory CD8^+^ T cells in lymphoid and mucosal tissues is not yet known; to address this, we first asked whether Bcl11b contributes to the clonal expansion of VacV-specific effector CD8^+^ T cells. Because Bcl11b is expressed by multiple cell types, we generated Cko mice by crossing mice expressing the codon-improved Cre recombinase (iCre) under the control of the Lck distal promoter (dLck-iCre) with mice homozygous for *lox*P-flanked allele of *Bcl11b* (*Bcl11b^flox/flox^*) (24, 25). In the resultant *Bcl11b^flox/flox^*/dLck-iCre^+^ mice, Bcl11b is expressed normally in CD4^+^CD8^+^ double-positive thymocytes as well as in CD4^+^ and CD8^+^ single-positive thymocytes; therefore, thymic T cell development is not affected in this system (24). However, the floxed Bcl11b allele is removed in ~85–90% of peripheral CD8^+^ T cells (24). For simplicity, the *Bcl11b^flox/flox^*/dLck-iCre^+^ mice will be referred to as *Bcl11b*^−/−^ Cko mice and *Bcl11b^flox/flox^*/dLck-iCre^−^ littermate controls will be referred to as WT mice.

**Figure 1:**
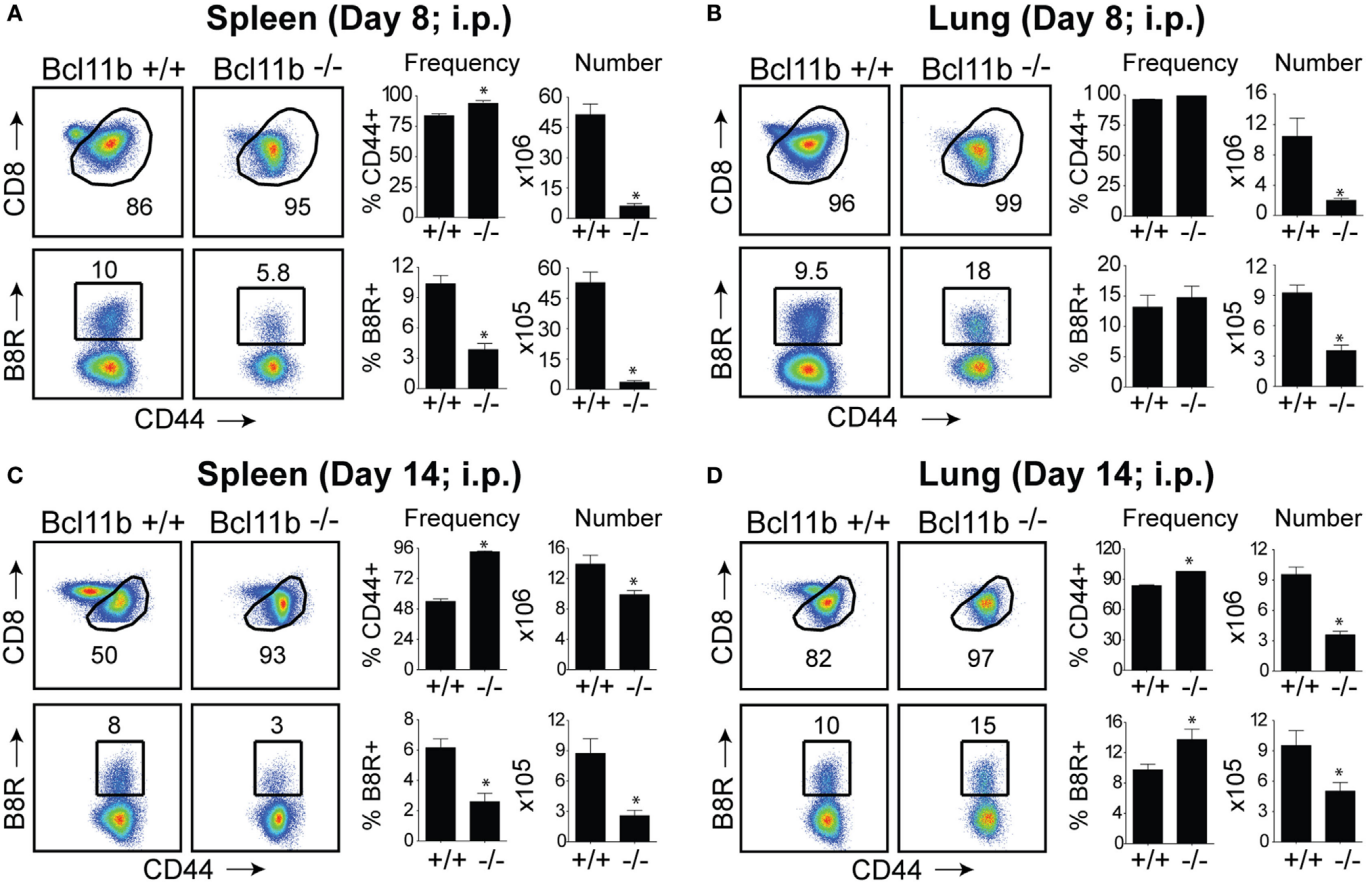
**Bcl11b is required for optimal accumulation of VacV-specific effector CD8 T cells**. WT (*Bcl11b^flox/flox^*/dLck-iCre^−^; *n* = 8) or Bcl11b-conditional knockout (*Bcl11b^flox/flox^*/dLck-iCre^+^; *n* = 7) mice were infected i.p with VacV-WR (2 × 10^5^ PFU/mouse). Eight **(A,B)** and fourteen **(C,D)** days postinfection, splenocytes and lung cells were harvested and stained for CD8, CD44, and B8R_20–27_/k^b^ tetramer. **(A–D)** *Left*, representative plots of CD8/CD44 (*Top Panels*) and CD8^+^CD44^hi^ B8R_20–27_/k^b^-tetramer staining (*Bottom Panels*), gating on live cells, are shown. Percentages of activated (CD44^hi^) and B8R_20–27_/k^b^ tetramer + CD8 T cells within each gate are indicated. *Right*, percentages and total numbers of CD8^+^CD44^hi^ and B8R_20–27_/k^b^ tetramer + cells per spleen **(A,C)** and lung **(B,D)**. Quadrant settings were based on controls, after gating on naïve CD44^lo^ cells in the same host. The results shown are representative of two separate experiments each with three to four mice per group. Asterisks indicate statistical significance. The *p*-values are <0.05 by two-tailed Student *t*-test (WT vs. Cko).

In initial experiments, we focused on systemic priming so it would not provide a bias to generating mucosa-associated CD8^+^ T cells. Cohorts of naïve, *Bcl11b*^−/−^ Cko, and WT littermate control mice were infected intraperitoneally (i.p.) with a sub-lethal inoculum of VacV-WR. On day 8, the reported peak of the primary effector response (1, 12), we tracked VacV-specific CD8^+^ T cells in the spleen and lung through the use of H-2K^b^ tetramers loaded with the immunodominant peptide epitope of VacV B8R protein (B8R_20–27_/k^b^) (30). In WT control mice, VacV infection induced a vigorous expansion of CD8^+^ T cells (Figures 1A,B). Notably, both the percentages and absolute numbers of B8R_20–27_/k^b^-reactive effector population in the spleen and lung corresponded closely to those obtained in non-transgenic WT C57BL/6 mice (1, 12). In stark contrast, total numbers of activated (CD44^hi^) and B8R_20–27_/k^b^-reactive CD8^+^ T cells were significantly reduced in both tissues sampled from *Bcl11b*^−/−^ Cko mice (Figures 1A,B). This was not due to impaired activation of CD8^+^ T cells in that CD44 (Figures 1A,B) and CXCR3 (Figure S1 in Supplementary Material) were similarly elevated in *Bcl11b*^−/−^ Cko mice. Downregulation of CD62L was also not different between *Bcl11b*^−/−^ Cko and WT cells (not shown). Impaired accumulation of VacV-specific CD8 T cells in the absence of Bcl11b was also observed on day 14, indicating that the defect cannot be explained by delayed kinetics of expansion (Figures 1C,D).

**Figure 2:**
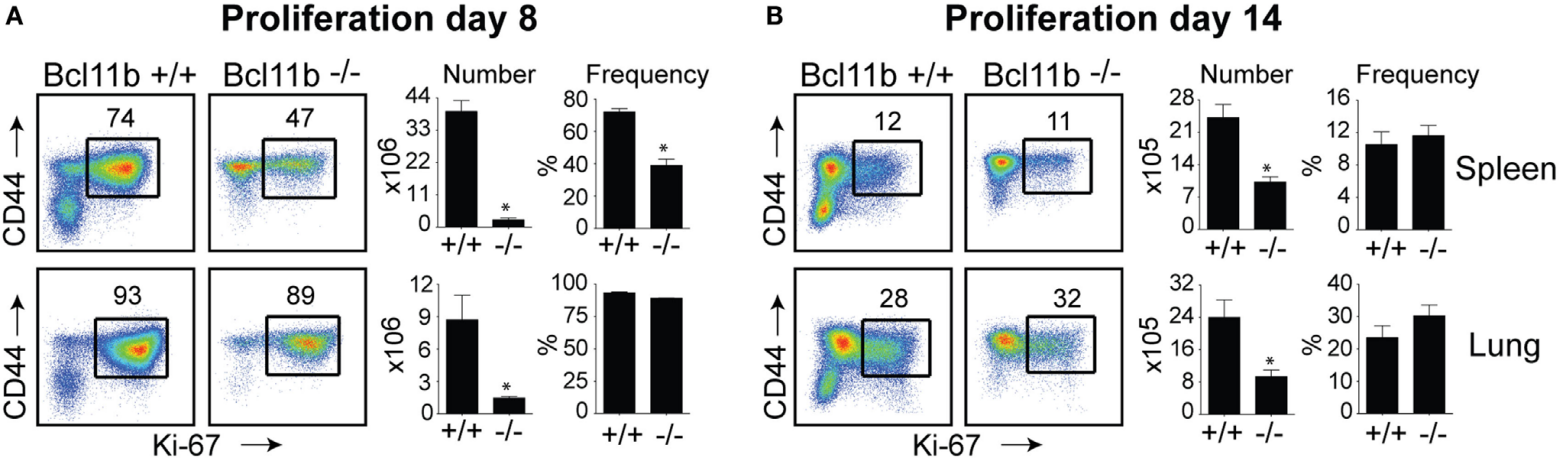
**Bcl11b controls the magnitude of expansion of virus-specific CD8 T cells in response to VacV-WR infection**. WT (*Bcl11b^flox/flox^*/dLck-iCre^−^; *n* = 8) or Bcl11b-conditional knockout (*Bcl11b^flox/flox^*/dLck-iCre^+^; *n* = 7) mice were infected i.p with VacV-WR (2 × 10^5^ PFU/mouse). Eight **(A)** and 14 **(B)** days postinfection, splenocytes and lung cells were harvested and stained for CD8, CD44, and Ki67. **(A,B)** *Left*, Representative plots showing the percentage of CD8 T cell proliferation by Ki67 staining among CD44^hi^ cells in VacV-infected mice. *Right*, total numbers and percentages of CD8^+^CD44^hi^Ki67^+^ cells per spleen and lung. Quadrant settings were based on controls, after gating on naïve CD44^lo^ cells in the same host. Percentages that stained positive for each marker are indicated. The results shown are representative of two separate experiments each with three to four mice per group. Asterisks indicate statistical significance. The *p*-values are <0.05 by two-tailed Student *t*-test (WT vs. Cko).

In the spleen of infected *Bcl11b*^−/−^ Cko mice, activated CD8^+^ T cells that stained positive for the proliferation marker Ki67 were reduced by 70–95% (Figures 2A,B), supporting our prior data that Bcl11b regulates proliferation of T cells responding to *Listeria monocytogenes* and influenza PR8 strain (24). Interestingly, percentages of CD8^+^CD44^hi^ T cells capable of proliferating (Ki67^+^) in response to VacV were not reduced in the lungs of *Bcl11b*^−/−^ Cko mice (Figures 2A,B; *Bottom Panels*). However, the reduced numbers of virus-specific CD8^+^ T cells generated translated into significant deficiencies in the accumulation of Ki67^+^ effector T cells (Figures 2A,B), indicating that Bcl11b might additionally influence the survival of cells that are destined to accumulate in the lungs.

### Bcl11b-Deficient CD8^+^ T Cells Adopt a Terminally Differentiated Effector Phenotype

**Figure 3:**
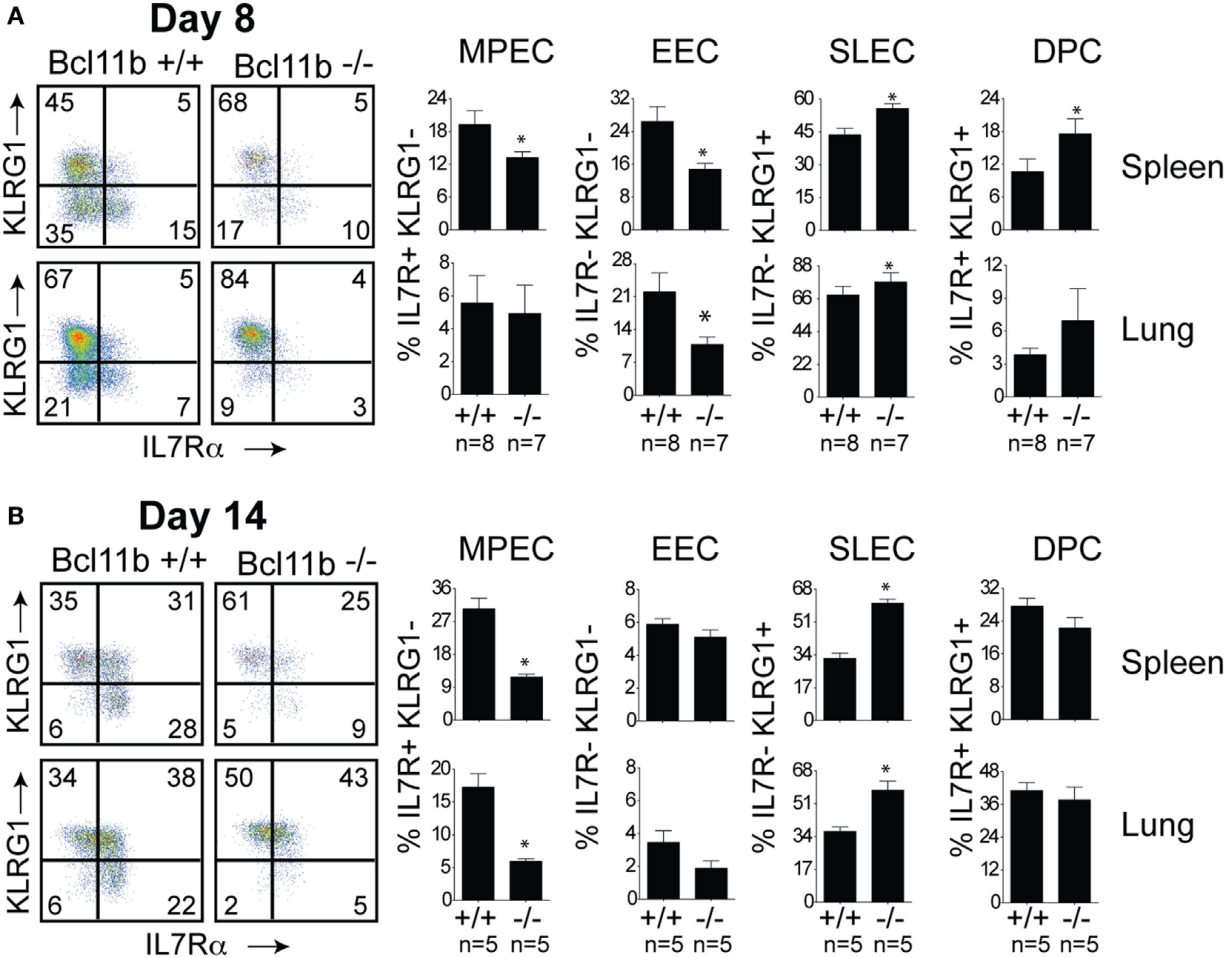
**Enhanced effector phenotype and loss of memory precursor effector CD8 T cells in the absence of Bcl11b**. WT (*Bcl11b^flox/flox^*/dLck-iCre^−^; *n* = 8) or Bcl11b-conditional knockout (*Bcl11b^flox/flox^*/dLck-iCre^+^; *n* = 7) mice were infected i.p with VacV-WR (2 × 10^5^ PFU/mouse). Eight **(A)** and 14 **(B)** days postinfection, splenocytes and lung cells were harvested and stained for CD8, CD44, IL-7Rα, KLRG1, and B8R_20–27_/k^b^ tetramer. **(A,B)** CD8 T cell differentiation was assessed with upregulation of KLRG1 and downregulation of IL-7Rα (CD127) on B8R_20–27_/k^b^ tetramer^+^ cells. Quadrant settings were based on controls, after gating on naïve CD44^lo^ cells in the same host. Percentages that stained positive for each marker are indicated. The results shown are representative of two separate experiments each with three to four mice per group. Asterisks indicate statistical significance. The *p*-values are <0.05 by two-tailed Student *t*-test (WT vs. Cko).

Next, we assessed whether the defective accumulation of virus-specific CD8^+^ T cells in infected tissues reflected a reduction in total effector cell numbers or a decrease in a specific stage of differentiation in response to VacV infection. To determine this, B8R_20–27_/k^b^-reactive cells were examined for expression of the co-inhibitory receptor killer-cell lectin-like receptor G1 (KLRG1) and IL-7 receptor alpha-chain (IL-7R) (19). Four major maturation and functional effector cell subsets were identified: a MPEC population characterized as KLRG1^lo^IL-7R^hi^ (Figures 3A,B, *lower right quadrants*); an early effector cell (EEC) population defined as KLRG1^lo^IL-7R^lo^ (Figures 3A,B, *lower left quadrants*); a terminally differentiated KLRG1^hi^IL-7R^lo^ SLEC population (Figures 3A,B, *upper left quadrants*); and a terminally differentiated KLRG1^hi^IL-7R^hi^ double-positive cell (DPC) (Figures 3A,B, *upper right quadrants*). During the expansion (day 8; Figure 3A) and contraction (day 14; Figure 3B) phases of the response, in *Bcl11b*^−/−^ Cko mice, the response to VacV was dominated by SLEC generation with relatively few MPECs and EECs present within the effector pool. Thus, during the priming phase of naïve CD8^+^ T cells, Bcl11b assimilates TCR and proinflammatory signals, ensuring proper expansion of effector T cells while restraining their commitment toward the SLEC lineage.

### Bcl11b Is Necessary for Acquisition of Optimal Effector Function by CD8^+^ T Cells

**Figure 4:**
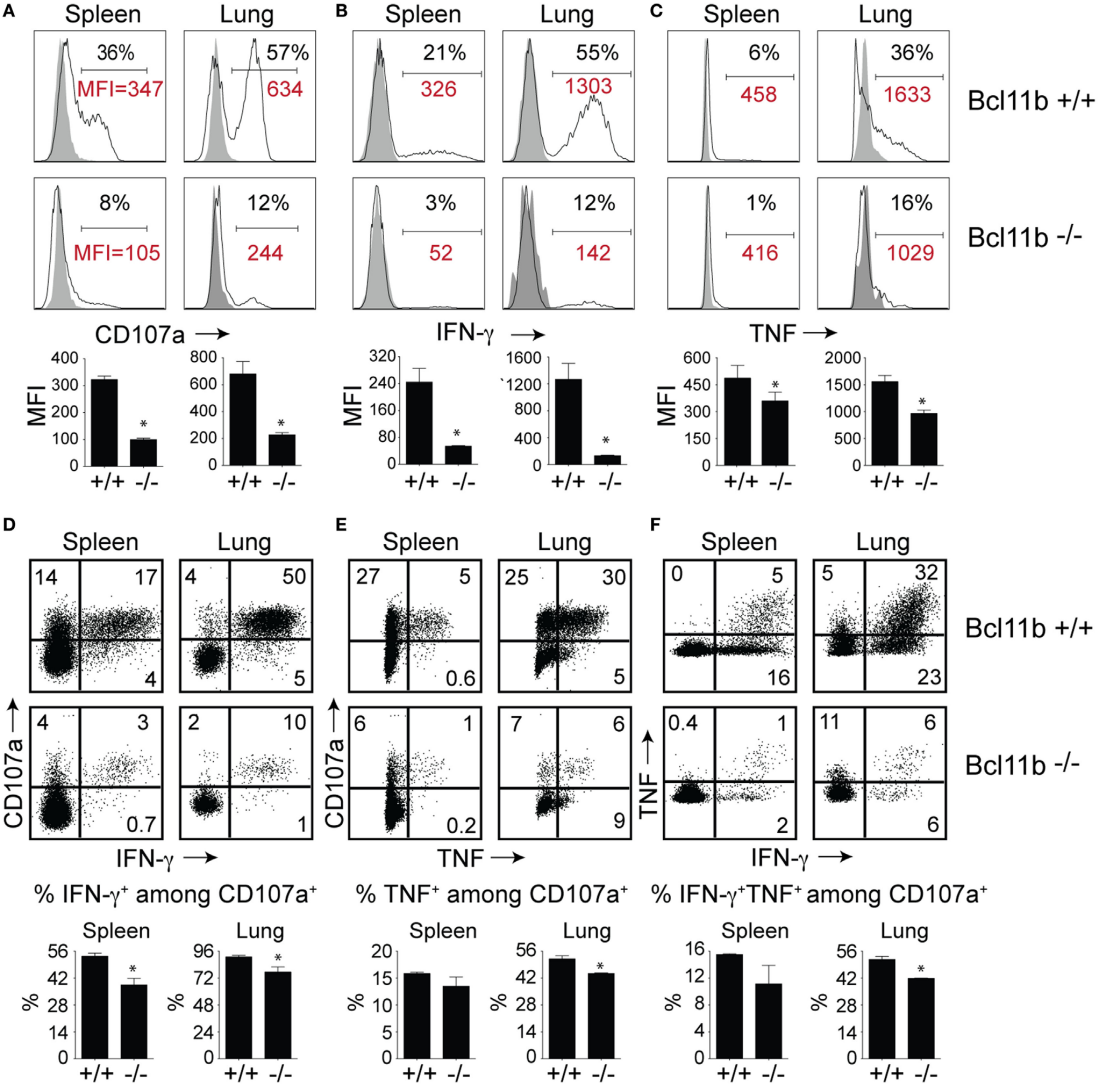
**CD8 T cells lacking Bcl11b are defective in lytic activity and anti-viral cytokine production after systemic infection with VacV**. WT (*Bcl11b^flox/flox^*/dLck-iCre^−^; *n* = 8) or Bcl11b-conditional knockout (*Bcl11b^flox/flox^*/dLck-iCre^+^; *n* = 7) mice were infected i.p with VacV-WR (2 × 10^5^ PFU/mouse). Eight days postinfection, splenocytes and lung cells were harvested and stimulated with B8R peptide for 8 h and subsequently stained for CD8α, CD107a, and intracellular IFN-γ and TNF. Representative plots for CD107a **(A)**, IFN-γ **(B)**, TNF **(C)**, CD107a/IFN-γ **(D)**, CD107a/TNF **(E)**, and IFN-γ/TNF **(F)** staining in gated CD8^+^CD44^+^ T cells. Numbers indicate percentages of positive cells or mean fluorescence intensity (MFI in red) within the gated population. Gates were based on controls, after gating on CD44^lo^ cells in the same host as well as using infected cells that were not stimulated with peptide and uninfected cells stimulated with B8R peptide for cytokine analysis (data not shown). The results shown are representative of two separate experiments each with three to four mice per group. The *p*-values are <0.05 by two-tailed Student *t*-test (WT vs. Cko).

High KLRG1 expression is a useful marker of multifunctional CD8^+^ T cells with potent cytolytic activity and the capacity to produce large quantities of anti-viral cytokines. Because Bcl11b deficiency was associated with acquisition of SLEC (KLRG1^hi^) phenotype, we hypothesized it might also be associated with enhanced expression of cytolytic effector molecules by virus-specific effector CD8^+^ T cells. First, we examined the expression of the lysosomal-associated membrane protein-1 (LAMP-1 or CD107a), which appears on the surface of CD8^+^ T cells when they release their pre-formed cytolytic granules (containing granzyme and perforin) in response to recognition of virus-infected cells and, therefore, is widely used as a marker of degranulation and cytolytic activity (33). On day 8 of infection, total spleen and lung cells were stimulated directly *ex vivo* for 8 h with the immunodominant VacV-derived peptide epitope, B8R (Figure 4A), or subdominant A8R peptide epitope (Figure S2 in Supplementary Material). As expected in WT mice, a large fraction of spleen (35–40%) and lung (50–60%) VacV-reactive CD8^+^ T cells expressed surface CD107a after peptide stimulation (Figure 4A and Figure S2 in Supplementary Material), indicating that extensive degranulation had occurred within the responding population. Remarkably, however, the majority of CD8^+^ T cells from *Bcl11b*^−/−^ Cko mice failed to degranulate following *ex vivo* peptide stimulation (Figure 4A). This observation was reflected in both the percentages (Figure 4A) and absolute numbers (not shown) of CD107a-positive effector cells present in the lung and spleen of infected mice. Furthermore, using mean fluorescence intensity (MFI) analysis, we found reduced levels of surface CD107a on *Bcl11b*^−^*^/^*^−^ Cko CD8^+^ T cells (Figure 4A), with an average MFI reduction between 40 and 70%. These results demonstrate that *Bcl11b*^−^*^/^*^−^ Cko effector CD8^+^ T cells produced fewer cytolytic effector molecules on a per-cell basis.

To further investigate the effects of Bcl11b expression, we analyzed CD8^+^ T cells for their capacity to produce IFN-γ and TNF following B8R (Figure 4B) or A8R (Figure S2 in Supplementary Material) peptide stimulation. Both peptides readily induced IFN-γ (Figure 4B and Figure S2 in Supplementary Material) and TNF (Figure 4C and Figure S2 in Supplementary Material) production in lung and splenic effector cells recovered from WT infected mice. However, relatively few IFN-γ- and TNF-expressing CD8^+^ cells could be detected in *Bcl11b*^−/−^ Cko mice (Figures 4B,C). In agreement with our CD107a data, the overall amount of IFN-γ and TNF produced on a per-cell basis (MFI) was lower in *Bcl11b*^−/−^ cells than in their WT counterparts.

Next, costaining for CD107a and IFN-γ or TNF was performed on VacV-reactive effector CD8^+^ T cells to determine if Bcl11b differentially impacted subsets within the CD107a^+^ population. In the WT spleen, ~55% of CD107a^+^ cells expressed IFN-γ (Figure 4D) and 15% expressed TNF (Figure 4E), whereas in the lung, ~95% of CD107a^+^ cells expressed IFN-γ. Bcl11b deficiency reduced the frequency of CD8^+^ CD107a^+^ T cells that produced IFN-γ and to a lesser extent TNF (Figures 4D,E; upper right quadrants). Similar results were found for CD8^+^ CD107a^+^ T cells that produced both IFN-γ and TNF (Figure 4F). Overall, these data demonstrate that in VacV-specific effector CD8^+^ T cells, Bcl11b is part of a transcriptional program that represses the formation of SLECs and simultaneously enhances lytic activity as well as expression of two major proinflammatory anti-viral cytokines, IFN-γ and TNF.

### Bcl11b Controls CD8 T Cell Effector Function Independently of T-Bet and Eomes

**Figure 5:**
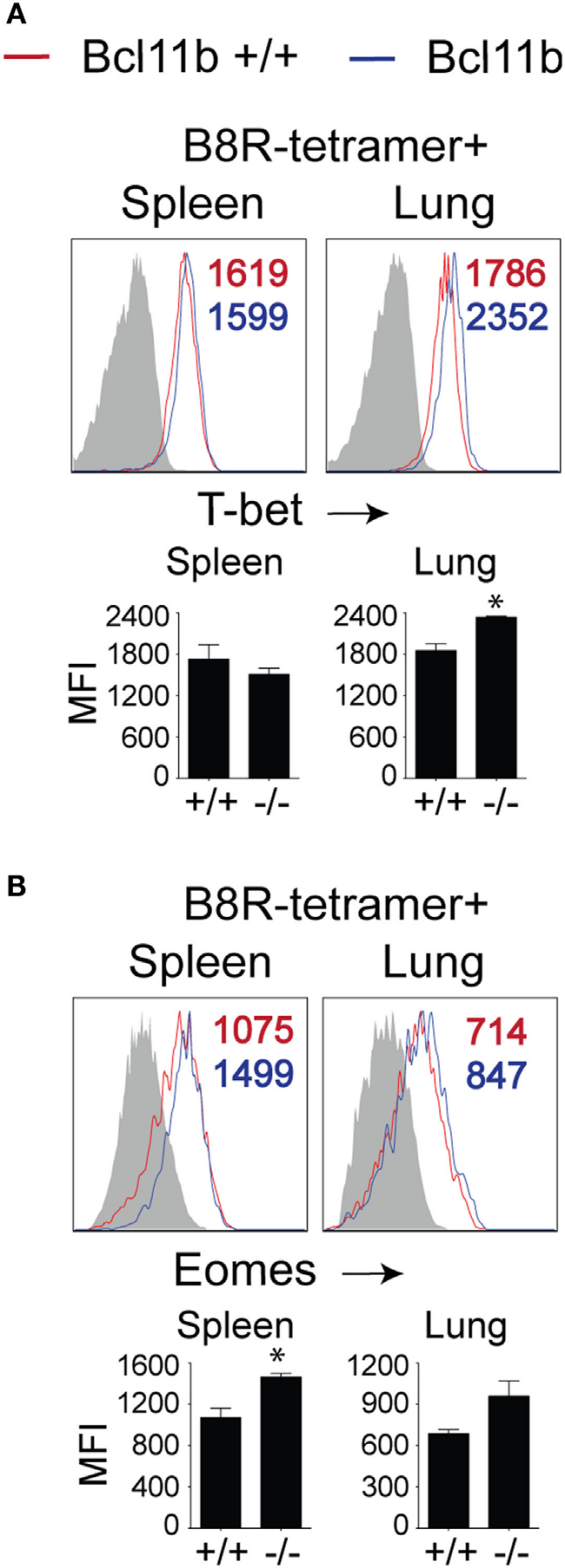
**Bcl11b deficiency does not affect T-bet and Eomes expression levels in VacV-specific CD8 T cells**. WT (*Bcl11b^flox/flox^*/dLck-iCre^−^) or Bcl11b-conditional knockout (*Bcl11b^flox/flox^*/dLck-iCre^+^) mice were infected i.p with VacV-WR (2 × 10^5^ PFU/mouse). Eight days postinfection, splenocytes and lung cells were harvested and subsequently stained for CD8α, CD44, T-bet, Eomes, and B8R_20–27_/k^b^ tetramer. Representative plots for T-bet **(A)** and Eomesodermin **(B)** staining gated on CD8^+^CD44^+^ B8R_20–27_/K^b^-tetramer^+^ cells recovered from the spleen and lung as indicated. Numbers indicate mean fluorescence intensity (MFI) within the gated population. Gates were based on controls, after gating on CD44^lo^ cells (Gray shaded area) in the same host. The *p*-values are <0.05 by two-tailed Student *t*-test (WT vs. Cko).

To date, several transcription factors have been identified as being critical for effector vs. memory CD8^+^ T cell differentiation in both viral and intracellular bacteria models of infection. Of crucial importance for SLEC differentiation is T-bet (19, 34, 35). Conversely, T-bet’s homolog, Eomes controls IL-15-dependent memory CD8^+^ T cell formation through directly activating *Il2rb* transcription (17). In addition, Eomes and T-bet cooperate to induce expression of Ifng, GzmB, and perforin and, thus, CTL effector function (16). As Bcl11b influenced MPEC/SLEC fate decision and function during VacV infection, we speculated that it might play a role in the balance of T-bet and Eomes in effector CD8^+^ T cells. Analysis of B8R_20–27_/k^b^-tetramer^+^ cells in both the spleen and lung showed that nearly all WT effector CD8^+^ T cells had upregulated T-bet (Figure 5A) and Eomes (Figure 5B) at the peak of the VacV response. Most strikingly, Bcl11b deficiency did not cause a decrease in the frequencies of B8R_20–27_/k^b^-reactive, T-bet^+^ CD8^+^ T cells compared with WT cells recovered from the spleen. Of note, in the lung, T-bet MFI in *Bcl11b*^−/−^ cells was slightly higher than WT cells, in line with their more pronounced terminally differentiated SLEC phenotype (Figure 5A). Similarly, we found that Bcl11b deficiency in CD8^+^ T cells resulted in a modest but reproducible increase in Eomes expression (Figure 5B). Together, these results provide evidence that the markedly impaired VacV-induced degranulation and anti-viral cytokines production by *Bcl11b*^−/−^ CD8 T cells was not caused by inadequate induction or altered balance between T-bet and Eomes. This implies important non-redundant roles for Bcl11b in mediating cytolytic activity and production of IFN-γ and TNF by VacV-specific CD8^+^ T cells.

Next, we considered the possibility that difference in viral load between WT and *Bcl11b*^−/−^ mice might have dramatic consequence on the differentiation and function of VacV-specific CD8 T cells, possibly leading to an exhausted phenotype. However, analysis of viral titers in the ovaries during both the acute (day 8) and contraction (day 14) phases of the response did not reveal any significant differences in the kinetics of clearance in WT and *Bcl11b*^−/−^ mice (Figure S3 in Supplementary Material). This is consistent with results in the i.p. infection model showing that depletion of CD8 T cells has no major effect on initial VacV-WR titers (10).

### Impaired Generation of Memory CD8^+^ T Cells in the Absence of Bcl11b

**Figure 6:**
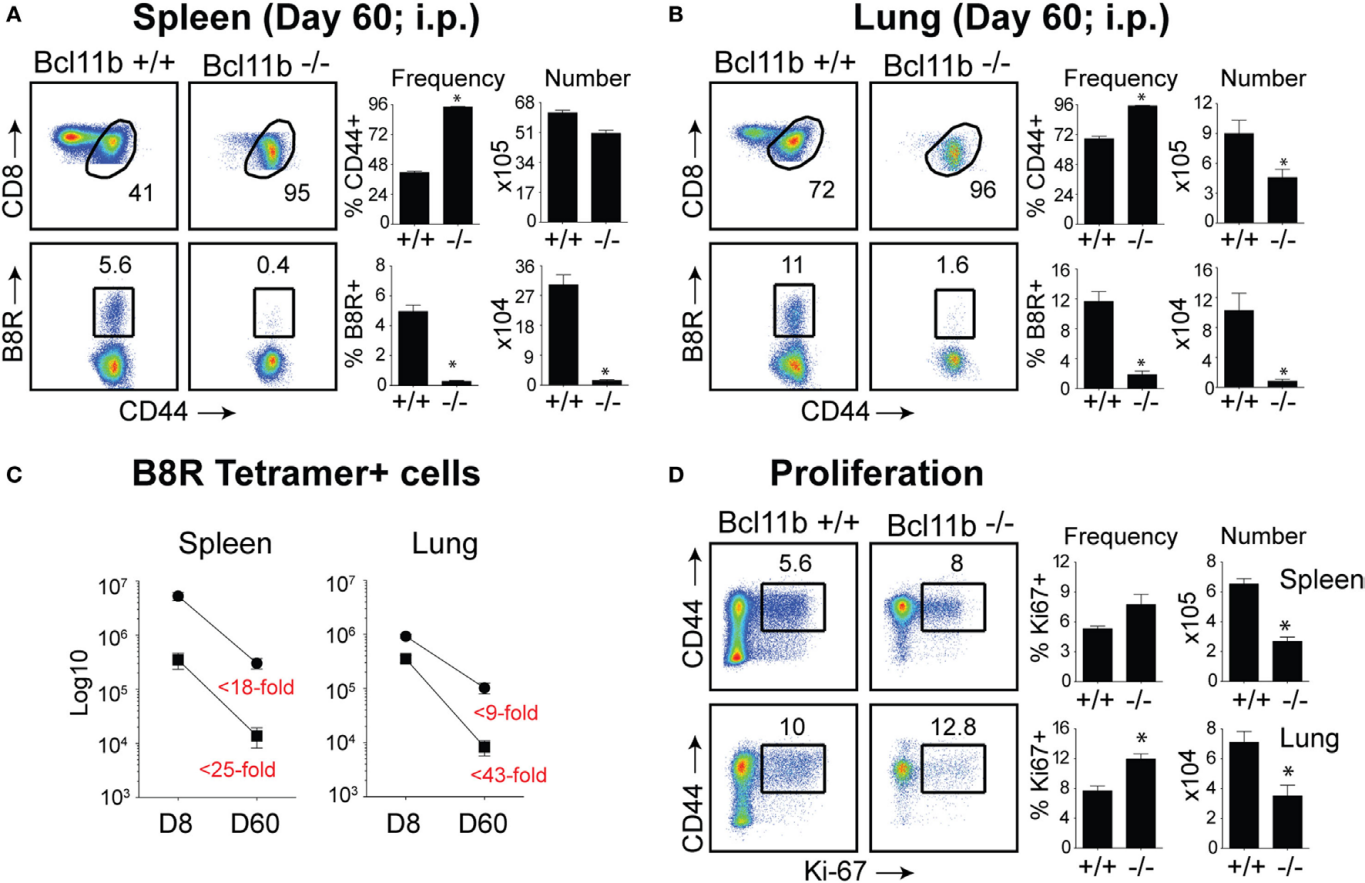
**Bcl11b is required for optimal generation of memory CD8 T cells after systemic infection with VacV**. WT (*Bcl11b^flox/flox^*/dLck-iCre^−^) or Bcl11b-conditional knockout (*Bcl11b^flox/flox^*/dLck-iCre^+^) mice were infected i.p with VacV-WR (2 × 10^5^ PFU/mouse). Sixty days postinfection, splenocytes and lung cells were harvested and stained for CD8, CD44, Ki67, and B8R_20–27_/k^b^ tetramer. **(A,B)** *Left*, representative plots of CD8/CD44 (*Top Panels*) and CD8^+^CD44^hi^ B8R_20–27_/k^b^-tetramer staining (*Bottom Panels*), gating on live cells, are shown. Percentages of activated (CD44^hi^) and B8R_20–27_/k^b^ tetramer + CD8 T cells within each gate are indicated. *Right*, percentages and total numbers of CD8^+^CD44^hi^ and B8R_20–27_/k^b^ tetramer + cells per spleen **(A)** and lung **(B)**. **(C)** Total numbers of B8R_20–27_/k^b^ tetramer + cells in spleen and lung at day 8 and 60 post infection. Numbers in red indicate the fold-decrease of response between days 8 vs. 60 within each group. **(D)** *Left*, representative plots showing the percentage of CD8 T cell proliferation by Ki67 staining among CD44^hi^ cells in VacV-infected mice. *Right*, Percentages and total numbers of CD8^+^CD44^hi^Ki67^+^ cells per spleen and lung. Quadrant settings were based on controls, after gating on naïve CD44^lo^ cells in the same host. Percentages that stained positive for each marker are indicated. The results shown are representative of two separate experiments each with four mice per group. Asterisks indicate statistical significance. The *p*-values are <0.05 by two-tailed Student *t*-test (WT vs. Cko).

Our data thus far demonstrated that lack of Bcl11b in virus-specific CD8^+^ T cells results in an altered SLEC/MPEC ratio, with effector cells skewing more toward SLEC lineage. Therefore, our next set of experiments was designed to assess whether *Bcl11b*^−/−^ effector CD8^+^ T cells would persist into the memory pool. Sixty days post infection, VacV-infected WT mice contained high frequencies of CD44^hi^ memory CD8^+^ T cells specific for the B8R epitope, regardless of whether cells were recovered from the spleen (Figure 6A) or lungs (Figure 6B), although lungs contained higher percentage of B8R-reactive population. In *Bcl11b*^−/−^ Cko mice compared with WT mice, we observed no significant differences in total CD8^+^ T cell numbers (not shown) and only a modest reduction in total CD44^hi^ cell numbers. By contrast, very few B8R_20–27_/k^b^-reactive memory CD8^+^ T cells were formed in *Bcl11b*^−/−^ Cko mice, irrespective of tissue sampled (Figures 6A,B). This may be, in large part, due to the reduced numbers of MPECs found in the absence of Bcl11b during the acute phase of the primary response to VacV, but might also reflect accelerated contraction and/or defective maintenance of *Bcl11b*^−/−^ CD8^+^ T cells that follows after viral clearance. Indeed, the fold reduction in the number of B8R_20–27_/k^b^-reactive cells recovered from *Bcl11b*^−/−^ mice vs. WT mice was substantially more between days 8 and 60 (spleen: ~25 vs. 18-fold; Lung: ~43 vs. 9-fold) (Figure 6C), supporting the idea that Bcl11b might control survival of VacV-reactive MPECs beyond the phase of primary expansion. Interestingly, no significant difference between *Bcl11b*^−/−^ Cko and WT mice was seen regarding the percentage of VacV-specific Ki67^+^ cells at day 60 postinfection (75–80%; Figure 6D), indicating that the progressive decline in the numbers of memory cells might be related to a survival disadvantage more than a defect in cytokine-driven homeostatic proliferation.

### Bcl11b Drives Effector and Memory CD8^+^ T Cell Responses Following Respiratory VACV Infection

**Figure 7:**
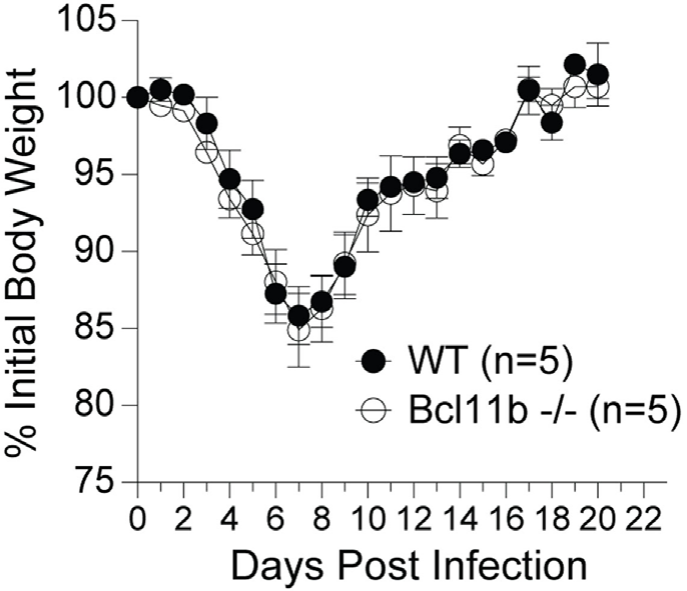
**Lack of Bcl11b in CD8 T cells does not alter disease symptoms, weight loss recovery, or survival after respiratory VacV-WR infection**. WT (*Bcl11b^flox/flox^*/dLck-iCre^−^) or Bcl11b-conditional knockout (*Bcl11b^flox/flox^*/dLck-iCre^+^) mice were infected i.n. with VacV-WR (1.5 × 10^4^ PFU/mouse). Animals were weighed daily and euthanized if weight loss was greater than 25% body weight. Mean% of initial body weight from indicated numbers of mice is shown.

**Figure 8:**
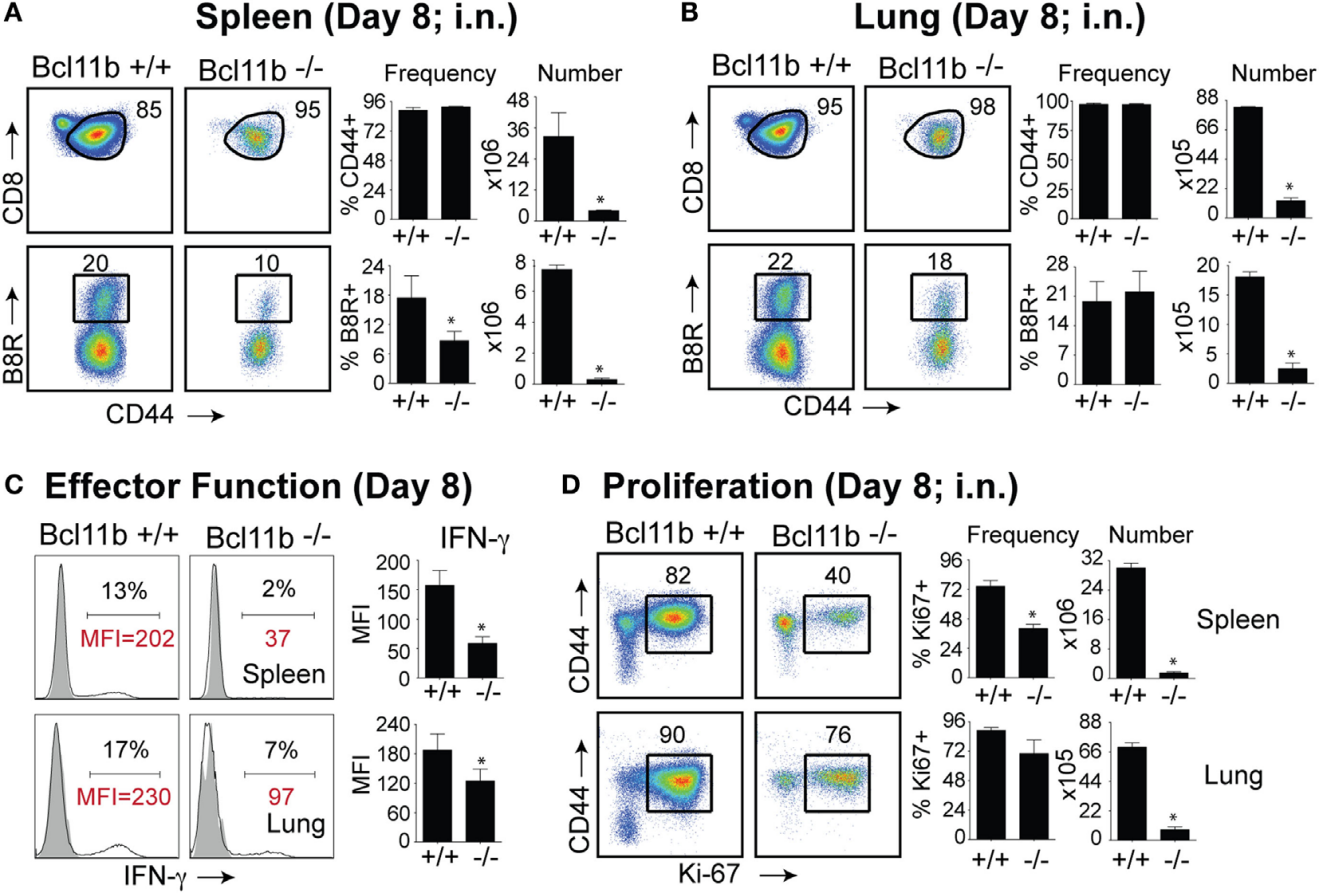
**Bcl11b expression is critical for the magnitude and functionality of primary effector CD8 T cells after respiratory infection with VacV**. WT (*Bcl11b^flox/flox^*/dLck-iCre^−^; *n* = 8) or Bcl11b-conditional knockout (*Bcl11b^flox/flox^*/dLck-iCre^+^; *n* = 8) mice were infected i.n. with VacV-WR (1.5 × 10^4^ PFU/mouse). Eight days postinfection, splenocytes and lung cells were harvested and stained for CD8, CD44, Ki67, and B8R_20–27_/k^b^ tetramer. **(A,B)** *Left*, representative plots of CD8/CD44 (*Top Panels*) and CD8^+^CD44^hi^ B8R_20–27_/k^b^-tetramer staining (*Bottom Panels*), gating on live cells, are shown. Percentages of activated (CD44^hi^) and B8R_20–27_/k^b^ tetramer + CD8 T cells within each gate are indicated. *Right*, percentages and total numbers of CD8^+^CD44^hi^ and B8R_20–27_/k^b^ tetramer + cells per spleen **(A)** and lung **(B)**. **(C)** Representative plots for intracellular IFN-γ staining in gated CD8+CD44+ T cells. Settings were based on controls, after gating on naïve CD44^lo^ cells in the same host. Percentages that stained positive for each marker are indicated. **(D)** *Left*, representative plots showing the percentage of CD8 T cell proliferation by Ki67 staining among CD44^hi^ cells in VacV-infected mice. *Right*, percentages and total numbers of CD8^+^CD44^hi^Ki67^+^ cells per spleen and lung. Settings were based on controls, after gating on naïve CD44^lo^ cells in the same host. Percentages that stained positive for each marker are indicated. The results shown are representative of two separate experiments each with four mice per group. Asterisks indicate statistical significance. The *p*-values are <0.05 by two-tailed Student *t*-test (WT vs. Cko).

**Figure 9:**
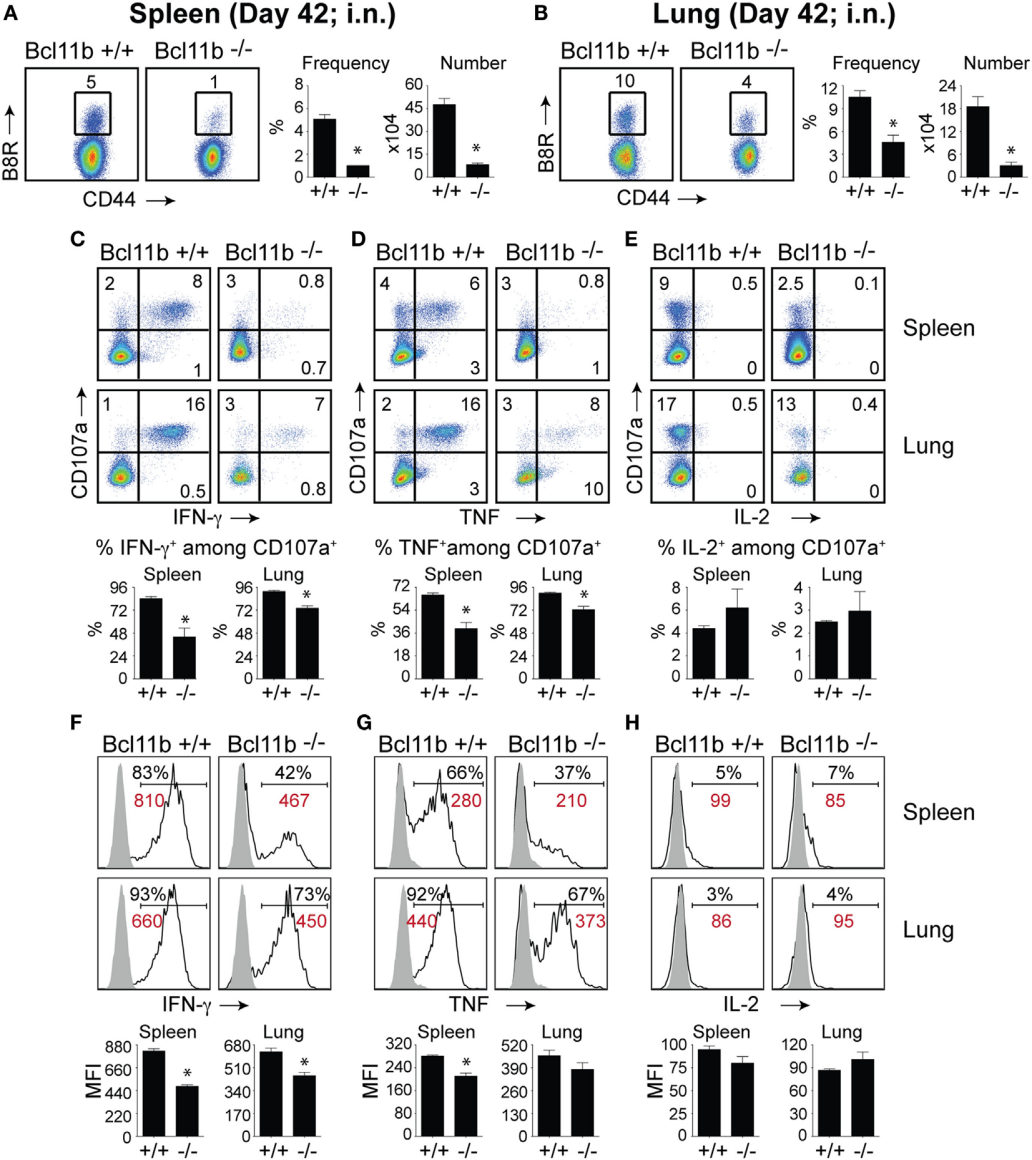
**Bcl11b expression is critical for the magnitude and functionality of memory CD8 T cells after respiratory infection with VacV**. WT (*Bcl11b^flox/flox^*/dLck-iCre^−^; *n* = 9) or Bcl11b-conditional knockout (*Bcl11b^flox/flox^*/dLck-iCre^+^; *n* = 9) mice were infected i.n. with VacV-WR (1.5 × 10^4^ PFU/mouse). Fourty-two days postinfection, splenocytes and lung cells were harvested and stained for CD8α, CD44, and B8R_20–27_/k^b^-tetramer **(A,B)** or stimulated with B8R peptide for 8 h and subsequently stained for CD8α, CD107a, and intracellular IFN-γ, TNF, and IL-2 **(C–H)**. **(A,B)** *Left*, representative plots of CD8^+^CD44^hi^ B8R_20–27_/k^b^-tetramer staining after gating on live cells. Percentages of B8R_20–27_/k^b^ tetramer + CD8 T cells within each gate are indicated. *Right*, Frequencies and total numbers (bar graphs) of B8R_20–27_/k^b^ tetramer + cells per spleen **(A)** and lung **(B)**. Representative dot plots (*Top Panels*) for CD107a/IFN-γ **(C)**, CD107α/TNF **(D)**, and CD107α/IL-2 **(E)** staining in gated CD8^+^CD44^+^ T cells. Bar graphs of the percentages (*Bottom Panels*) of each cytokine within CD8^+^CD44^+^CD107a^+^ fraction are shown. Representative histogram plots (*Top Panels*) for IFN-γ **(F)**, TNF **(G)**, and IL-2 **(H)** gated on CD8^+^CD44^+^CD107a^+^ cells recovered from the spleen and lung as indicated. Numbers indicate percentages of positive cells or mean fluorescence intensity (MFI in red) within the gated population. Gates were based on controls (filled gray), after gating on CD44^lo^ cells in the same host. Percentages that stained positive for each marker are indicated. Bar graphs of the MFI (*Bottom Panels*) of each cytokine within CD8^+^CD44^+^CD107a^+^ fraction are shown. The results shown are representative of two separate experiments each with three to four mice per group. Asterisks indicate statistical significance. The *p*-values are < 0.05 by two-tailed Student *t*-test (WT vs. knockout).

It is noteworthy that we observed a strong reduction in virus-specific effector and memory CD8^+^ T cells in the lungs, even though the animals were infected via i.p. injection. This raised the possibility that Bcl11b mediated signals might promote mucosal memory. To address this more directly, we infected WT and *Bcl11b*^−/−^ Cko mice intranasally with a sub-lethal inoculum of VacV-WR and analyzed the effector and memory responses in these mice 8 and 42 days following infection. Bcl11b deficiency in CD8 T cells did not modify the kinetics of the initial weight loss (Figure 7; days 0–7), recovery (Figure 7; days 7–20), or survival profiles of VACV-infected mice. However, correlating with our data in the i.p. infection model, at the peak of the T cell response (day 8), we observed markedly reduced numbers of total activated (CD44^hi^) and B8R_20–27_/k^b^-reactive CD8^+^ T cells in the lung (Figure 8A) and spleen (Figure 8B) of *Bcl11b*^−/−^ Cko mice compared with WT mice, and the IFN-γ response in both tissues was dramatically reduced (Figure 8C). As in the i.p. infection model, proliferation of CD8^+^ T cells in the spleen was reduced in the absence of Bcl11b, whereas proliferative responses of mucosal effector CD8^+^ T cells were largely unaffected (Figure 8D). Most significantly, however, 15- to 20-fold fewer B8R_20–27_/k^b^-reactive memory CD8^+^ T cells were present in the spleen (Figure 9A) and lungs (Figure 9B) 42 days after infection of *Bcl11b*^−/−^ mice vs. WT mice. Thus, Bcl11b confers competitive fitness to memory cells located in the lung regardless of whether infection is via the peritoneal cavity or respiratory tract.

Again, both the percentages and MFI of IFN-γ- and TNF-positive CD8 T cells present in the lung and spleen of VACV-infected *Bcl11b*^−/−^ mice were reduced compared with WT mice (Figures 9C,D,F,G). We previously found that Bcl11b directly regulates IL-2 in CD4^+^ T lymphocytes (36). Since IL-2 is important for formation of memory CD8 T cells, we examined intracellular IL-2 expression in the lung and spleen of WT and *Bcl11b*^−/−^ mice. In contrast to expression of IFN-γ, we found that loss of Bcl11b had little or no effect on the ability of VacV-specific CD8 T cells to produce IL-2 in response to peptide stimulation (Figures 9E,H). Thus, Bcl11b may act to control the formation and/or survival of memory cells independently of IL-2.

## Discussion

Here, we provide evidence that absence of Bcl11b during the immune response to VacV infection caused increased differentiation of SLECs, while MPEC frequency was dramatically reduced. Interestingly, although the frequency of SLECs was increased in the absence of Bcl11b, these cells had reduced ability to produce IFN-γ and degranulate. Importantly, the few MPECs that formed in the absence of Bcl11b failed to accumulate over time and populate the memory pool in both lymphoid and mucosal tissues. Thus, Bcl11b is an important regulator of MPEC vs. SLEC fate decision and, consequently, memory cell potential in virus-specific CD8^+^ T cells.

An important observation in the current study is that *Bcl11b*^−/−^ CD8^+^ T cells showed reduced capacity to produce IFN-γ and degranulate after peptide stimulation, even in the presence of normal levels of T-bet and Eomes. This was unexpected given that T-bet is essential for cytotoxic activity and optimal production of IFN-γ by VacV-specific CD8^+^ T cells (37). Currently, we do not know why T-bet and/or Eomes cannot function in the absence of Bcl11b but we are actively pursuing these lines of investigations. In this regard, we previously found that Bcl11b associates with genomic regions upstream of Gzmb and perforin transcriptional start site and enhanced the ability to trigger Ag-dependent killing by CD8^+^ T cells (24). Thus, the simplest explanation may be that T-bet requires Bcl11b to exert its full activity on the cytotoxic gene expression and effector function. Additionally, Bcl11b might be required to cooperate or regulate other transcription factors critical for CTL function, such as Blimp-1, recently found to be needed by T-bet for full CTL activity in the response to LCMV (38). T-bet and Blimp-1 are both required for SLEC differentiation, in addition to CTL activity, however, Bcl11b negatively regulates this process, while sustaining the CTL activity, thus playing a unique role in CD8^+^ T cells. How Bcl11b restricts SLEC differentiation but sustains CTL function remains to be established.

Most strikingly, absence of Bcl11b caused diminished formation of MPECs and ablated memory cell development in lung and lymphoid organs. Other transcription factors that control MPEC formation include Eomes, a T-box transcription factor highly homologous to T-bet and expressed in activated CD8^+^ T cells (17). Eomes controls IL-15-dependent memory CD8^+^ T cell formation through directly activating *Il2rb* transcription (17). Similar to T-bet, we found the protein levels of Eomes were not altered in VacV-specific *Bcl11b*^−^*^/^*^−^ CD8^+^ T cells, suggesting that Bcl11b may act independently of Eomes in regulating the development of memory cells. Future studies should attempt to identify downstream targets of Bcl11b in CD8^+^ T cells and determine whether it can interact with or regulate other fate-determining transcription factors.

Two other transcription factors, Id2 and Id3, known to negatively regulate the DNA-binding activity of E-proteins, were recently found to control the differentiation of SLECs and MPECs, respectively (39, 40). IL-2, IL-12, and IL-21 enhance Id2 expression in antigen-specific CD8^+^ T cells, while decreasing Id3 expression (39). Id2 was found to control SLEC survival through Bim repression, and globally the transcriptional program of SLECs, including cytokine expression (39, 40). Thus, it is possible that Bcl11b may work in concert with Id3 to generate MPECs and memory CD8^+^ T cells, while suppressing Id2 in restricting the SLEC program. FOXO1, a transcription factor inhibited by AKT signaling, plays a critical role in the formation of memory CD8^+^ T cells through the upregulation of memory signature transcription factors, such as Eomes, TCF7/TCF-1, and Id3, and the repression of terminal effector signature transcription factors, such as Blimp-1, T-bet, and Id2 (41). In this network of transcription factors, which regulate SLEC vs. MPEC fate, we established so far that T-bet and Eomes are not downstream of Bcl11b, however it is of great interest to determine whether Bcl11b is regulated by T-bet and/or Eomes and/or cooperates with them. Additionally, it would be important to further establish the position of Bcl11b in the network of transcription factors that regulate SLECs and MPECs and particularly the relationship with TCF7, Id2, Id3, Bcl-6, Blimp-1, and FOXO1.

## Conclusion

Our previous study (24) together with those shown here suggest that the critical requirement for Bcl11b for proliferative responses of CD8^+^ T cells in lymphoid tissues may be a general phenomenon that applies to a variety of microbial pathogens administered via different routes. Furthermore, they support a model in which Bcl11b promotes the memory potential in effector CD8^+^ T cells through enhancing survival and functionality of memory precursors, and in parallel, suppresses the differentiation into SLECs. A better understanding of Bcl11b function at discrete stages of the immune response could facilitate future development of novel vaccines that aim to generate long-lasting protective T cell memory against blood-borne and mucosal pathogens.

## Author Contributions

GA, JS, VT, and PD designed and performed experiments, analyzed and interpreted data, and contributed to the writing of the manuscript. TH performed plaque assays, analyzed and interpreted data. KL assisted with mouse genotyping and colony maintenance. JC assisted with mouse genotyping and colony maintenance and writing of the manuscript. DA interpreted data and wrote the manuscript. SS-A conceived and designed experiments, analyzed and interpreted data, wrote the manuscript, and supervised the study. All authors read and approved the final manuscript.

## Conflict of Interest Statement

The authors declare that the research was conducted in the absence of any commercial or financial relationships that could be construed as a potential conflict of interest.

## Abbreviations

Ag, antigen; Cko, conditional knockout; Eomes, eomesodermin; LCMV, lymphocytic choriomeningitis virus; MPECs, memory precursor effector cells; SLECs, short-lived effector cells; VacV, vaccinia virus; WR, western reserve; WT, wild type.
