# Supplementary figures and images for: Transcription Factor Bcl11b Controls Effector and Memory CD8 T cell Fate Decision and Function during Poxvirus Infection
Source: Front Immunol. 2016 Oct 13;7:425. doi: 10.3389/fimmu.2016.00425 (PMC5061747; doi:10.3389/fimmu.2016.00425)

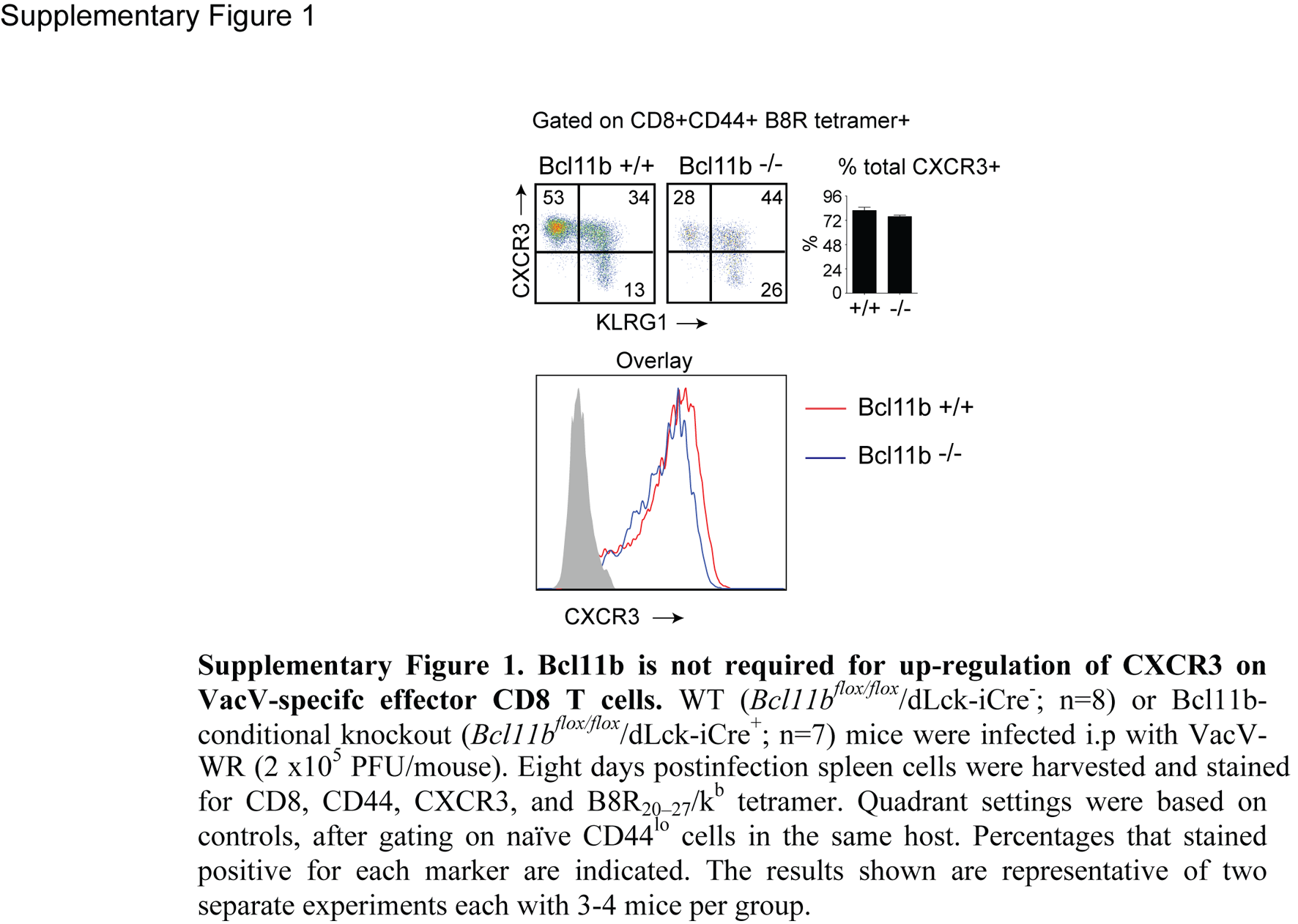

Supplement: Supplementary file 1 [file Image_1.tif]

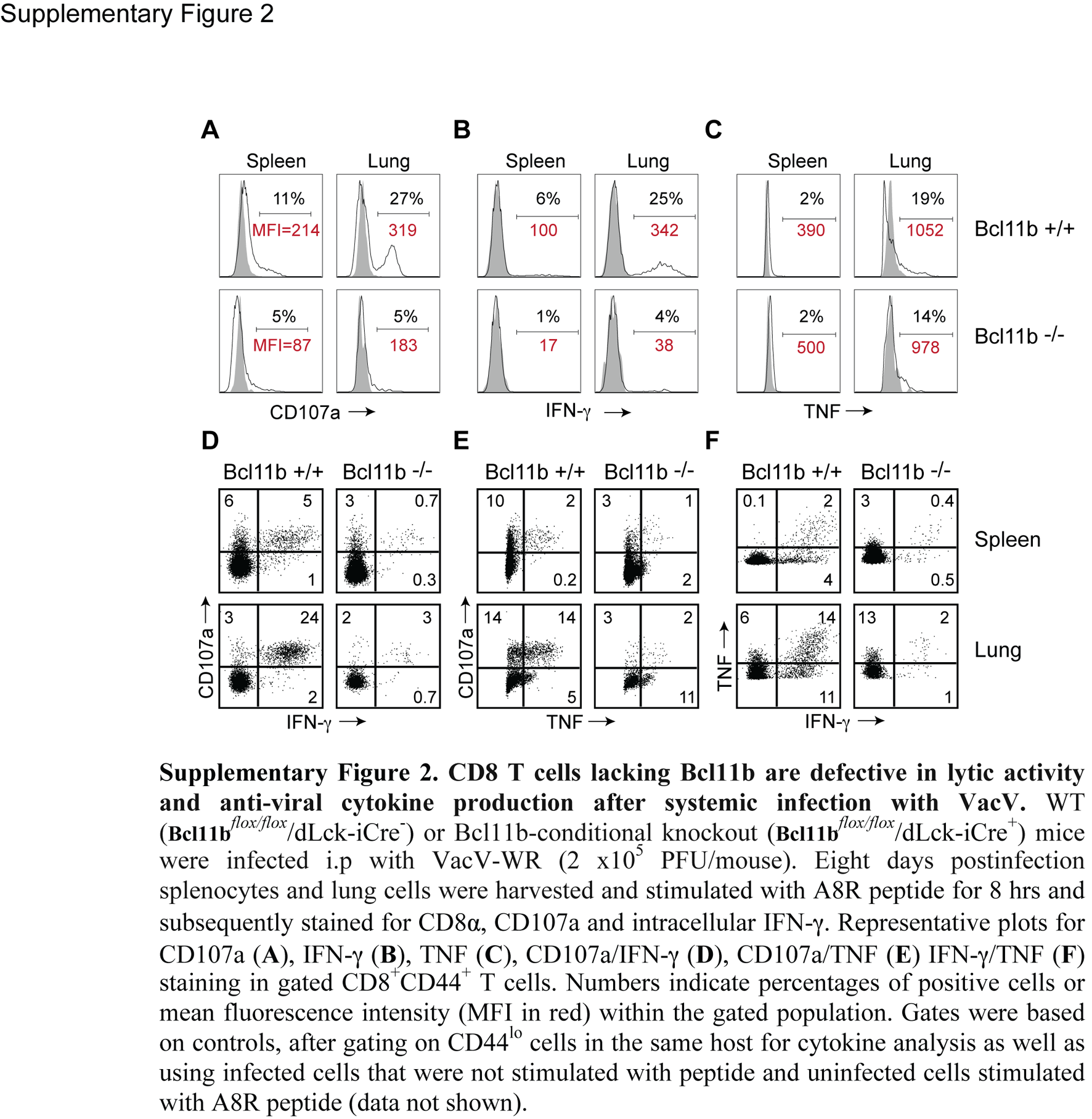

Supplement: Supplementary file 2 [file Image_2.tif]

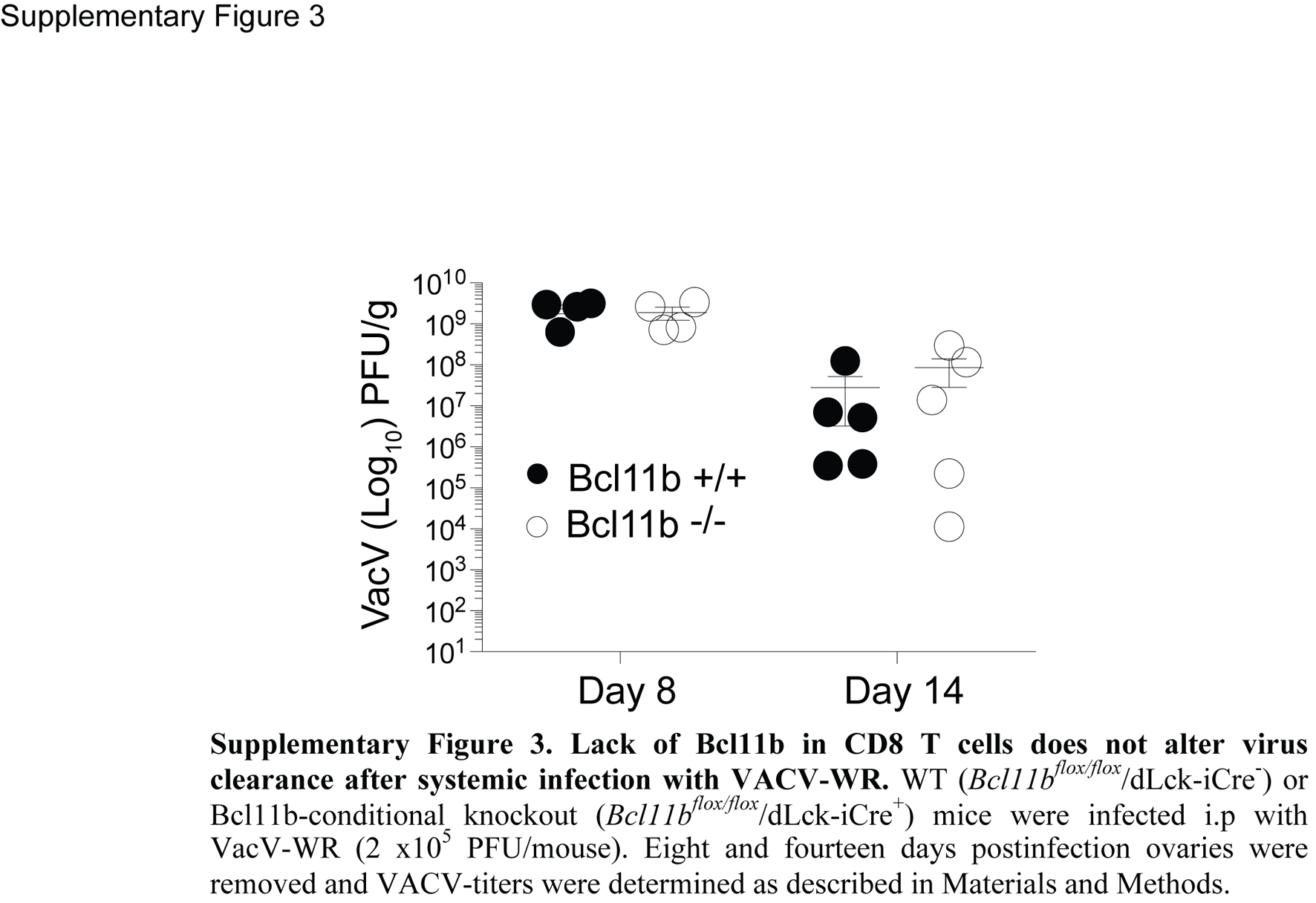

Supplement: Supplementary file 3 [file Image_3.tif]
